# Tumor Progression Locus 2 (Tpl2) Kinase as a Novel Therapeutic Target for Cancer: Double-Sided Effects of Tpl2 on Cancer

**DOI:** 10.3390/ijms16034471

**Published:** 2015-02-25

**Authors:** Hye Won Lee, Han Yong Choi, Kyeung Min Joo, Do-Hyun Nam

**Affiliations:** 1 Department of Neurosurgery, Samsung Medical Center, Sungkyunkwan University School of Medicine, 135-710 Seoul, Korea; E-Mail: nsproper@naver.com; 2 Department of Urology, Samsung Medical Center, Sungkyunkwan University School of Medicine, 135-710 Seoul, Korea; E-Mail: hanyong.choi@samsung.com; 3 Department of Health Sciences and Technology, SAIHST, Sungkyunkwan University, 135-710 Seoul, Korea; 4 Department of Anatomy and Cell Biology, Samsung Medical Center, Sungkyunkwan University School of Medicine, 135-710 Seoul, Korea

**Keywords:** tumor progression locus 2, cot, mitogen-activated protein kinase kinase kinase 8, cancer, oncogene, tumor suppressor gene, therapeutic target

## Abstract

Tumor progression locus 2 (Tpl2) is a mitogen-activated protein kinase (MAPK) kinase kinase (MAP3K) that conveys various intra- and extra-cellular stimuli to effector proteins of cells provoking adequate adoptive responses. Recent studies have elucidated that Tpl2 is an indispensable signal transducer as an MAP3K family member in diverse signaling pathways that regulate cell proliferation, survival, and death. Since tumorigenesis results from dysregulation of cellular proliferation, differentiation, and apoptosis, Tpl2 participates in many decisive molecular processes of tumor development and progression. Moreover, Tpl2 is closely associated with cytokine release of inflammatory cells, which has crucial effects on not only tumor cells but also tumor microenvironments. These critical roles of Tpl2 in human cancers make it an attractive anti-cancer therapeutic target. However, Tpl2 contradictorily works as a tumor suppressor in some cancers. The double-sided effects of Tpl2 originate from the specific upstream and downstream signaling environment of each tumor, since Tpl2 interacts with various signaling components. This review summarizes recent studies concerning the possible roles of Tpl2 in human cancers and considers its possibility as a therapeutic target, against which novel anti-cancer agents could be developed.

## 1. Introduction

**Figure 1:**
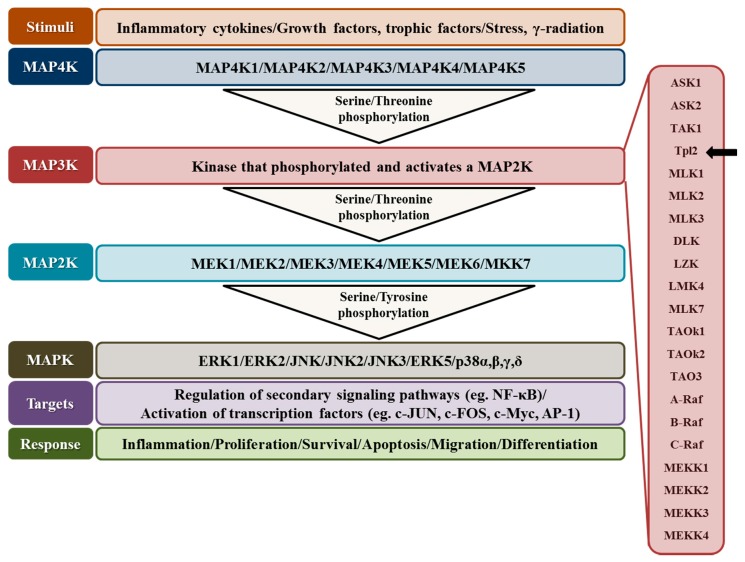
The typical MAPK signaling pathways mediating the transmission of external signals to intra-cellular effector proteins. MAP4K: mitogen-activated protein kinase kinase kinase kinase; MAP3K: mitogen-activated protein kinase kinase kinase; MAP2K: mitogen-activated protein kinase kinase; MAPK: mitogen-activated protein kinase.

The mitogen-activated protein kinase (MAPK) cascades are evolutionarily conserved signaling pathways in eukaryotic cells. They especially regulate cell proliferation, survival, and death in response to a wide range of extra-cellular signals including growth factors, cytokines, and physical/chemical stresses [1,2]. The typical MAPK signaling cascade is composed of 3–5 kinase families (MAPK kinase kinase kinase (MAPKKKK/MAP4K), MAPK kinase kinase (MAPKKK/MAP3K), MAPK kinase (MAPKK/MAP2K), MAPK, and MAPK-activated protein kinases (MAPKAPK)). Each kinase family phosphorylates and activates the next kinase family [3,4,5] (Figure 1). More specifically, the cascade that leads to activation of MAPKs requires a dual specific phosphorylation on serine/tyrosine residues of MAPKs by MAP2Ks and on Serine/Threonine residues of MAP2Ks by MAP3Ks. Well-ordered activation of numerous MAPKs in a cell is regulated by MAP3Ks which provide stimulus- and cell-specific signaling contexts [6]. Substrates of MAPKs include MAPK-activated protein kinases (MAPKAPK) family, which comprises the ribosomal-S6-kinases (RSK1–4), the MAPK-interacting kinases (MNK1 and 2), the mitogen-and stress-activated kinases (MSK1 and 2), and MAPKAPKs MAPKAPK-2 (MK2), MAPKAPK-3 (MK3), and MAPKAPK-5 (MK5) [7,8,9,10,11]. Upon activation of MAPKs, they provoke intra-cellular effects mainly through phosphorylation of target proteins, which in turn alters various functional characteristics of target proteins such as stability, DNA binding, intra-cellular localization, protein-protein interaction, and post-translational modification [1,2]. Additionally, MAPKs can regulate DNA transcription non-enzymatically [12]. On the other hand, the atypical MAPK pathways include the extracellular signal-regulated kinases ERK3, ERK4, ERK7, ERK8, and nemo-like kinase (NLK) which can converge to different MAPK-activated protein kinases (MAPKAPK) such as mitogen-activated protein kinase-activated protein kinase-5 (MK5) MK5 [13].

## 2. Structure-Based Activation Mechanism of Tumor Progression Locus 2 (Tpl2/Cot/MAP3K8)

MAP3Ks provide specific activation of MAP2K-MAPK pathways through unique interactions with upstream and downstream signaling effectors [6]. Tumor progression locus 2 (Tpl2/Cot/MAP3K8) is a member of the MAP3K serine/threonine protein kinase family, which includes 21 characterized MAP3Ks [14] (Figure 1). Tpl2 was initially cloned as a proviral integration locus of Moloney Murine Leukemia Virus (MoMuLV) in murine T-cell lymphoma cells [15,16,17,18,19,20,21]. The encoded protein contains a serine/threonine kinase domain, an amino-terminal region with unknown function, and a carboxy-terminus that carries a “degron” sequence (amino acid (a.a.) 435–457). The “degron” sequence is important for the protein stability of Tpl2 [6]. Carboxy (*C*)-terminal truncations of Tpl2 (Tpl2ΔC) that occur as a result of provirus insertion in MoMuLV-induced T-cell lymphomas and Mouse mammary tumor virus (MMTV)-induced mammary adenocarcinomas in rats remove this “degron” sequence to reduce proteasomal degradation of Tpl2 [6,16,22]. In comparison to wild-type (WT) Tpl2, expression of Tpl2ΔC was associated with elevated catalytic activity and wider signaling capacity [17]. In addition, *C*-terminus of Tpl2 has important phosphorylation sites critical for Tpl2 activation of ERK-1/2 [23,24,25], and is one of the major binding sites for the nuclear factor κ-light-chain-enhancer of activated B cells (NF-κB) precursor protein p105 and A20-binding inhibitor of NF-κB-2 (ABIN2) [26].

An important aspect of Tpl2 activation is its interaction with upstream components of the nuclear factor κ-light-chain-enhancer of activated B cells (NF-κB) pathway, such as the inhibitor of κ B (IκB) kinase β (IKKβ/IKK2) [27,28,29]. In the steady state, the entire pool of Tpl2 is held in complex with ABIN2 and p105. This interaction stabilizes Tpl2 but also prevents Tpl2 and NF-κB from activating their downstream signaling cascades by inhibiting the kinase activity of Tpl2 and the proteolysis of NF-κB precursor protein p105 [28,30,31]. Upon activation of Tpl2 by various stimuli, IKK phosphorylates p105/NF-κB1 at Ser927 and Ser932, which flags p105 for proteasomal degradation and releases Tpl2 from the complex [32,33]. The newly liberated Tpl2 phosphorylates its substrates but is unstable to be targeted for proteasome-mediated degradation, thus restricting prolonged activation of Tpl2 and its downstream signaling pathways [31,34]. Furthermore, dissociated p105/NF-κB1 is proteolysed into p50, which dimerizes with other NF-κB family members, translocates into the nucleus, and regulates the transcription of over 400 genes [6,30].

There are two Tpl2 isoforms; 58 kDa (Tpl2 long) and 52 kDa (Tpl2 short) [6]. Three ATGs at the 5' end of Tpl2 mRNA give rise to 467-, 464-, and 438-a.a. proteins. The 467- and 464-a.a. proteins are detected as a 58-kDa band (p58), whereas the 438-a.a. protein is detected as a 52-kDa (p52) band in western blot analysis [14]. Although the p58 and p52 isoforms are expressed at similar levels in most tissue types, the 58-kDa isoform is predominant when Tpl2 is overexpressed in 293 cells. A recent study has shown that phosphorylation at Thr-290 plays an obligatory role in Tpl2 activation by external signals [31,35,36,37]. Both p52 and p58 isoforms of Tpl2 form a stable complex with p105 in the resting state. However, the p58 isoform is preferentially phosphorylated at Thr-290 by stimulation, which is more efficient when p58 is binding to p52 [34]. Furthermore, the p58 isoform is released from p105 preferentially upon stimulation compared with the p52 isoform [34]. The released p58 is active but undergoes rapid proteasome-mediated degradation, which is dependent on the phosphorylation at Thr-290 [34]. 

## 3. Tpl2 Kinase-Mediated Downstream Signal Transduction

There are four major MAPKs in mammals; extracellular signal-regulated kinases (ERKs), extracellular signal-regulated kinase 5 (ERK5), c-Jun *N*-terminal kinases (JNKs), and p38 MAPKs (Figure 1) [3,4]. In general, growth factors, cellular stresses, and either growth factors or stresses are considered as the main activator of ERK1/2, JNK/p38-MAPKs, and ERK5 cascade, respectively. However, accumulated evidence has suggested cross talks between various components of the pathways. These multiple interactions between the different MAPK cascades serve to provoke more diverse and precise cellular responses to various intra- and extra-cellular stimuli [5,38]. 

Tpl2 has a major role in the activation of ERK1/2 through its direct substrate, the mitogen-activated extracellular signal-regulated kinase (MEK, a MAP2K) [16,18]. However, Tpl2 can also activate JNK, to a lesser extent, p38, and ERK5 through direct phosphorylation of their upstream MAP2Ks; MKK4, MKK6, and MEK5, respectively [17,18,19,20,21,39,40]. Furthermore, DNA damage induced by Ultraviolet B (UVB) stimulates translocation of Tpl2 to the nucleus upon phosphorylation, where nuclear Tpl2 phosphorylates histone H3 at Ser10 to increase the transcriptional activity of c-Fos [41]. 

In the absence of extra-cellular signals, endogenous Tpl2 is inactive. However, when overexpressed, WT Tpl2 as well as oncogenic Tpl2ΔC is constitutively active in a variety of cell types [38,42,43,44,45,46,47,48]. They activate a plethora of signaling pathways to influence cell survival, proliferation, and transformation [48] in concert with other signaling molecules, such as MAPK [18,39,49], nuclear factor-activated T cells (NFAT), and NF-κB pathways [19,20,21,30]. However, these widespread effects of overexpressed Tpl2 may include overexpression artifacts, which need to be further validated by specific knockout studies for each downstream signaling component.

## 4. Functions of Tpl2 in the Immune System

At first, Tpl2 was reported to play its critical roles in the immune system [50,51]. Tpl2 is associated with numerous inflammatory pathways including ERK, JNK, p38, and NF-κB [14,43,52,53,54,55,56,57]. For example, Tpl2 regulates Tumor Necrosis Factor (TNF) synthesis through the ERK-mediated phosphorylation of the TNF-converting enzyme, TACE, on Thr735 [54]. Various pro-inflammatory stimuli such as lipopolysaccharide (LPS), TNF, and CD40 ligand activate Tpl2 through Toll-like receptors (TLRs), TNF receptor 1 (TNFR1), CD40, and interleukin 1 (IL-1) receptor [14,50,55,58,59]. 

The innate immune response, the first line of defense against infections, is orchestrated by macrophages, dendritic cells, natural killer cells, and neutrophils. The crucial initial step to provoke the innate immune response is the detection of pathogen-associated molecules by TLRs. TLRs activate the Tpl2-MEK-ERK pathway in macrophages, which regulates not only the production of cytokines but also the cellular responses to TNF, IL-1β, and CD40 ligand [60,61,62,63,64]. Production of prostaglandin E2 (PGE2) and its regulatory enzyme, COX-2, in monocytes are also regulated by the Tpl2-ERK-mitogen- and stress-activated protein kinase-1 (MSK1) pathway [65,66]. Furthermore, Tpl2 is required for the optimal induction of p38 MAPK in the stimulation of bone marrow-derived dendritic cells by LPS or CpG [43,67]. This suggests that Tpl2 transduces a broad inflammatory signal in innate immune cells.

In the adaptive immune system, Tpl2 is expressed in B and T lymphocytes [14,22,50]. The CD40-Tpl2-ERK signaling pathway mediates immunoglobulin isotype switching in B cells [14], insensitivity to LPS-mediated B cell apoptosis [59], and IL-12-mediated helper T cell differentiation [22,38]. For example, in a TNF-driven Crohn’s-like inflammatory bowel disease mouse model, the absence of Tpl2 reduced numbers of memory CD4^+^ and peripheral CD8^+^ lymphocytes and ameliorated the onset and progression of the disease [68].

Although previous studies using mouse inflammatory disease models suggest that Tpl2 has predominantly pro-inflammatory functions, in some circumstances, Tpl2 inhibits pro-inflammatory cytokines and functions in an anti-inflammatory manner [38,42]. For example, Tpl2 deficiency resulted in over-production of pro-inflammatory cytokines [43] and reduced synthesis of an anti-inflammatory cytokine, IL-10 [69]. In a mouse model of ovalbumin (OVA)-induced bronchoalveolar inflammation, Tpl2 ablation led to increase in both OVA-specific and total IgE and shifted the balance of sytokine production toward the Th2 cytokines [44]. Those contradictory roles of Tpl2 in the immune system indicate that Tpl2 can have ambivalent effects on cancer development and progression in a cell-type-specific manner.

## 5. Tpl2 in the Development and Progression of Human Cancers

**Figure 2:**
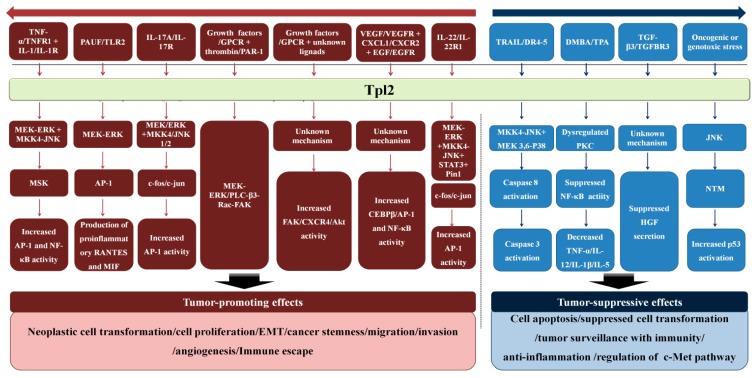
Tpl2 mediated signaling cascades involved in cancer development and progression.

The MAPK signaling pathway including Tpl2 regulates the development and progression of cancers [70]. Since Tpl2 interacts with many upstream and downstream signaling components, the roles of Tpl2 in human cancers is complex (Figure 2). Either over-expression or reduced-expression of this gene can promote tumorigenesis depending on cancer types [6] (Table 1). The complexity might originate from the specific extra- and intra-cellular signaling context of each human cancer. 

**Table 1:** Contradictory roles of Tpl2 kinase in cancer.

Tumor-Promoting	Tumor-Suppressive
Elevated Tpl2 activity was demonstrated in a number of human cancers and associated with tumorigenesis and cancer progression via activation of the MAPK signaling pathway [14,17,18,19,20,45,47,50,62 ,64,71,72,73,74,75,76,77]. Tpl2 promoted tumorigenesis and cancer progression via the phosphorylation of Pin1 resulting cyclin D1 up-regulation and induced EMT by IL-22/MEK/ERK, JNK/STAT3/AP-1 signaling pathway in breast cancer [62,64,78,79]. Tpl2 facilitated tumor growth by PAUF-mediated MEK/ERK signaling pathway, resulting in increasing expression of pro-tumorigenic cytokines [80]. Inhibiting Tpl2 significantly reduced peritoneal dissemination by inducing endoplasmic reticulum stress and inhibiting EMT and also blocked angiogenesis in gastric cancer [81,82]. Tpl2 transduces PAR1 signals to regulate the expression of MMPs and other secreted molecules both in fibroblasts and tumor cells, and to promote reorganization of the actin cytoskeleton and cell migration [74,83]. Tpl2 kinase contributes to disease progression of clear cell renal cell carcinoma through activated MAPK signaling and cross-talk with CXCL12/CXCR4-directed chemotaxis and chemoinvasion [84]. Elevated Tpl2 activity promoted CRPC growth via activation of MEK/ERK and NF-kB signaling pathway [85]. Tpl2 enhanced tumor progression and metastasis of CRPC through increased cell proliferation, stemness, migration and invasion abilities via activation of FAK/Akt and CXCL12/CXCR4 signaling pathways [86]. Tpl2 mediated TNF-α downstream signaling promoting cell survival, invasion, and angiogenesis [14,55,58,63,87]. Tpl2 plays an important role in promoting IL-17A-induced tumorigenesis in colon cancer, cervical cancer and breast cancer via IL-17A-activated Tpl2 signaling pathway to function upstream of MEK/ERK and JNK/c-Jun [88,89,90]. Tpl2 enhances cancer metastasis through versican/TLR2/6-Tpl2 mediated activation of myeloid cells in the metastatic niche [6,26,91,92,93].	Transduction of anti-proliferative T cell receptor signals and inhibitory effects on transformation of chronically stimulated T cells [75]. Tpl2 ablation significantly enhanced tumor initiation and progression in a chemical-induction mouse skin cancer model via up-regulated NF-κB signaling and MMP activity [94,95,96]. Tpl2 induced resistance to TRAIL induced apoptosis in breast cancer cells, reduced [97]. Tpl2 antagonized cell transformation and survival through JNK dependent up-regulation of NPM required for optimal p53 response to oncogenic or genotoxic stress in urethane-induced lung tumors in mice [98]. Tpl2 ablation promoted colitis-associated cancer through down-regulation of IL-10 and regulatory T-cell numbers in the intestinal mucosa and increased HGF secretion of intestinal myofibroblasts [69,99]. Human effector memory CD8^+^ cytotoxic T cells, which play a major role in adaptive anti-tumor immune responses, are regulated by the IL-12 induced Tpl2/MEK/ERK pathway [100].

## 6. Tpl2 as a Tumor-Promoting Oncogene

Tpl2 is expressed in most tissues but at relatively low levels [45,46]. Elevated Tpl2 activity was demonstrated in a number of human cancers including breast cancer, colon cancer, endometrial cancer, gastric cancer, nasopharyngeal carcinoma, thymoma, lymphoma, and EBV-related Hodgkin’s lymphoma [17,18,45,62,64,71,72,73]. Up-regulation of Tpl2 in various tumor types strongly indicates its association with tumorigenesis and/or cancer progression [14,19,20,47,50,73,74,75,76,77]. 

Tpl2 conveys various oncogenic signals in a variety of human solid tumors. Tpl2 expression is up-regulated in approximately 40% of human breast cancer specimens [62,64]. Since Tpl2 gene amplification was detected in eight out of the Tpl2-over-expressing 14 breast cancer specimens, increased number of Tpl2 gene could be a possible mechanism for Tpl2 over-expression [64]. Tpl2 directly interacts with Peptidyl-prolyl cis-trans isomerase NIMA-interacting 1 (Pin1) and induces the phosphorylation of Pin1 on Ser16, which results in cyclin D1 up-regulation and breast cancer development [78]. In addition, interleukin-22 (IL-22) phosphorylates Tpl2 through IL-22R1, activates the MEK-ERK, JNK, and STAT3 signaling pathways, and results in the epithelial-mesenchymal transition (EMT) through AP-1 in breast cancer [79]. 

There is considerable evidence on the role of Tpl2 in cancer metastasis. Proteinase-activated receptor 1 (PAR1) activated by several proteinases including thrombin and matrix metalloproteinase-1 (MMP-1) promotes cell transformation, tumor metastasis, and angiogenesis in a variety of cancers including prostate cancer and melanoma [101,102,103,104]. Tpl2 transduces the PAR1 signals to regulate the expression of MMPs and other secreted molecules both in fibroblasts and tumor cells [74]. Tpl2 is also required for PAR1 to engage a Rac1 and focal adhesion kinase (FAK)-dependent pathway, to activate ERK and JNK1, and to promote reorganization of the actin cytoskeleton and cell migration [74,83]. Pancreatic adenocarcinoma upregulated factor (PAUF), an endogenous ligand of TLR2 and TLR4, is overproduced in certain types of cancer including pancreatic cancers [105]. PAUF activatesTLR2-mediated TPL2-MEK-ERK signaling pathway to increase expression of pro-tumorigenic cytokines, but inhibits TLR-mediated NF-κB signaling, thereby facilitating tumor growth and escape from innate immune surveillance [80].

Peritoneal dissemination of cancer cells is closely associated with poor oncologic outcomes [65,66,67,106]. Angiogenic factors including vascular endothelial growth factor (VEGF) and chemokine (C-X-C motif) ligand 1 (CXCL1) are involved in this process [107,108,109,110]. VEGF and CXCL1 increase phosphorylation and kinase activity of Tpl2 in a dose- and time-dependent manner [81]. For example, in gastric cancer, Tpl2 inhibition significantly induced endoplasmic reticulum stress, inhibited EMT, and reduced peritoneal dissemination of cancer cells, which was accompanied by angiogenesis blockage [81,82]. These findings suggest that Tpl2 inhibitors could lead to the development of novel simultaneous anti-angiogenic and anti-metastatic treatment strategies. 

Recently, we further elucidated a novel function of Tpl2 kinase in the disease progression of genito-urinary cancers [94,95]. In clear cell renal cell carcinoma (ccRCC) with innate high metastatic ability, Tpl2 mRNA levels are significantly elevated compared with normal kidneys [6]. Its downstream signaling components, MKK and ERK, were also activated in human RCC cases [111,112]. In our study, we detected that up-regulation of Tpl2 is significantly associated with the presence of metastases and poor clinical outcomes in human ccRCCs [84]. Moreover, elevated Tpl2 activity enhanced tumorigenic and metastatic potential of ccRCC cells significantly in preclinical ccRCC models through activation of the MAPK signaling and cross talk with the CXCL12-CXCR4-directed chemotaxis and chemoinvasion [84]. 

The expression of TLR4 is closely associated with the severity of prostate cancer [113]. Tpl2 has an important role in the downstream MAPK signaling of TLRs under acute or chronic inflammatory conditions [14,50,55,58,59]. Chronic prostatic inflammation is a major inducing factor of prostate cancer, which supports the possible connection between the Tpl2 signaling pathway and the development of prostate cancer [114]. Importantly, Tpl2 was over-expressed in human castration resistant prostate cancer (CRPC) and Tpl2 drove CRPC growth through the MEK-ERK and NF-κB signaling pathway [85]. We further demonstrated that Tpl2 induces EMT and maintains stemness of CRPC cells, which increase proliferation, clonogenic, migration, and invasion abilities of CRPC cells significantly [86]. Although more detailed studies are necessary to elucidate the detailed mechanism, we found that FAK-Akt and CXCL12-CXCR4 axis may be alternative downstream signaling pathways activated by Tpl2 kinase in CRPC cells, through previous study [86]. 

## 7. Tpl2 as a Key Player in Inflammatory Cancer Microenvironment

Cancer-related inflammation aids proliferation and survival of malignant cells, stimulates angiogenesis and metastasis, inhibits adaptive immunity to tumor cells, and alters tumor-responses to anti-cancer therapies [115,116,117]. Activation of oncogenes or inactivation of tumor suppressor genes leads to alterations in nearby inflammatory cytokine network, which is of great importance in the processes of cancer-related inflammation [118]. Given the impact of Tpl2 on the innate and acquired immunity, Tpl2 could contribute to cancer progression and metastasis via tumor-associated inflammatory response.

Inflammatory cells in the tumor microenvironment produce TNFα, a major stimulator of Tpl2, which can promote cell survival, invasion, and angiogenesis [119,120,121,122,123,124,125,126,127,128]. Activation of theTpl2-MEK-ERK pathway in macrophages and monocytes positively regulates the production ofpro-inflammatory cytokines such as TNFα [14,55,58,63,87]. Interleukin-17A (IL-17A) is a pro-inflammatory cytokine that is expressed mainly by activated memory T-cell [129,130]. It activates NF-κB and AP-1 transcription factors through the IL-17 receptor [131,132]. In contrast to the restricted expression of IL-17A, the IL-17 receptor is expressed universally [130]. Recent studies suggested that Tpl2 plays an important role in IL-17A-induced tumorigenesis of colon cancer [88], cervical cancer [89], and breast cancer [90]. IL-17A-activated Tpl2 functioned as an upstream of MEK/ERK and JNK/c-Jun [90] to increase c-fos and c-jun transcriptional activity, to induce AP-1-dependent transcription, and ultimately to provoke cellular transformation [90]. Furthermore, versican, a large extra-cellular matrix proteoglycan, enhances cancer metastasis through TLR2/6-Tpl2 mediated activation of myeloid cells in the metastatic niche [6,26,91,92,93]. Accordingly, ablation of Tpl2 in an *in vivo* myeloma model led to prolonged disease latency via abrogation of the “inflammatory switch” in myeloma–associated monocytes/macrophages within nascent myeloma lesions [133]. 

## 8.Tpl2 as a Tumor-Suppressor Gene

In contrast with the strong evidence associated with the oncogenic roles of Tpl2, under certain conditions, Tpl2 may serve tumor suppressive roles. In one study, Tpl2 knockout (Tpl2^−/−^) mice, when crossed with mice with a T cell receptor transgene that provokes T cell lymphoma, showed a higher incidence of the tumor [75]. Moreover, the Tpl2^−/−^ mice had a significantly higher incidence of tumor initiation and faster malignant progression in a chemical-induction mouse skin cancer model [94,95]. Mechanistically, oncogenic effects of Tpl2 ablation were mediated by increased NF-κB activity, which ultimately induced both skin tumorigenesis and inflammation [95]. Comparative gene expression profiling between Tpl2^+/+^ and Tpl2^−/−^ mice further demonstrated that MMP1b/2/9/13 that stimulate the migration and invasion of cancer cells are up-regulated in Tpl2^−/−^ keratinocytes [96]. 

Tpl2 may have a broader role in dictating the balance between cell survival and death. Tumor protein p53 has a crucial role in tumor suppression, in part by regulating apoptosis. In breast cancer cells, reduced Tpl2 expression was associated with resistance to TNF-related apoptosis-inducing ligand (TRAIL)-induced apoptosis, a p53-independent process [97]. Tpl2 utilizes similar mechanisms in lung carcinogenesis and meets the requirement for a suppressor gene [98]. Low Tpl2 levels were correlated with reduced lung cancer patient survival and accelerated onset and multiplicity of urethane-induced lung tumors in mice. Tpl2 was found to antagonize oncogene-induced cell transformation and survival through JNK dependent up-regulation of nucleophosmin (NPM), which is required for the optimal p53 response to oncogenic or genotoxic stresses [98]. Collectively, these data indicate that Tpl2 is a positive regulator of the p53 pathway in human lung cancer. 

Tpl2 ablation also promoted intestinal inflammation through down-regulation of IL-10 levels and regulatory T-cell numbers in the intestinal mucosa of Tpl2^−/−^ mice. The inflammation was responsible for the tumorigenesis of colitis-associated cancer [69]. In addition, cell-specific ablation of Tpl2 in intestinal myofibroblasts (IMFs) developed significantly increased numbers and sizes of colon cancers, which were associated with enhanced epithelial proliferation and decreased apoptosis [99]. Tpl2-deficient IMFs up-regulated HGF production and became less sensitive to the negative feedback by TGF-β3. These results indicate that Tpl2 normally suppresses colon epithelial tumorigenesis through the negative regulation of the HGF-c-Met pathway. 

Finally, Tpl2 participates as an upstream of the MEK-ERK pathway in the positive regulation effects of IL-12 on the functions of human effector memory CD8^+^ cytotoxic T lymphocytes, which plays a major role in adaptive anti-tumor immune responses [100]. 

## 9. Development of Anti-Cancer Tpl2-Targeting Agents

Tpl2-targeting agents could be effective therapeutic strategies for many types of inflammatory disease including cancers since they have critical roles in the immune system [134]. Especially, Tpl2 has been an attractive target for anti-inflammatory drugs because it is activated selectively by inflammatory stimuli, however, it appears not to be crucial for the activation of ERK1/2 by the T cell and B cell receptors [135]. This could allow the adaptive immune system to provide protection against infection [135]. A few series of potent, reversible, ATP-competitive Tpl2 specific inhibitors, such as 1,7-naphthyridine-3-carbonitriles [136], 8-substituted-4-anilino-6-amiquionline-3-carbonitrile [137], thienopyridines [138], quinoline-3-carbonitriles [139], and the 4-alkylamino-1,7-naphthyridine-3-carbonitriles [140], were identified for immunologic purposes. More recently developed Tpl2 inhibitors showed improved specificity and anti-inflammatory efficacy [87,139,141,142,143,144].

Given the important roles of Tpl2 in tumorigenesis, metastasis, and cancer-related neo-angiogenesis, Tpl2-targeting agents may also provide novel insights into potential anti-cancer therapeutics strategies in several cancers in which Tpl2 plays an important role as a tumor promoting gene [134]. Moreover, Tpl2 acts on both tumor cells and inflammatory tumor microenvironments through diverse signaling pathways including the MAPK cascades, which would offer multi-modal therapeutic mechanisms. Besides the MAPK pathway, TNFα-Tpl2 mediated pathways could be additional therapeutic targets for developing anti-tumor agents since TNFα-mediated COX2 expression plays an important role in inflammation and carcinogenesis. For example, luteolin (2-(3,4-dihydroxyphenyl)-5,7-dihydroxy-4-chromenone), which inhibits Tpl2 activity by direct binding in an ATP-competitive manner, primarily targeted Tpl2 involved in cancer-associated inflammation and exerted potent anti-tumor activities [145]. Luteolin attenuated TNFα-induced COX-2 expression by down-regulating the transactivation of NF-κB and AP-1 [145]. 

However, any therapeutic use of kinase inhibitors should consider the cost-benefit ratio carefully. Although small molecule inhibitors exist for the targeting of the Tpl2-MEK-ERK pathway, side effects could be a serious issue. Consistent with the diminished chemokine receptor expression levels in both resting and activated Tpl2^−/−^ macrophages, Tpl2 ablation resulted in impaired *in vivo* macrophage recruitment to tissue sites of inflammation [146]. Although these findings provided scientific bases of how Tpl2 inhibition could provide an alternative treatment for a variety of autoimmune diseases, the physiological effect of Tpl2 ablation could significantly impair the immunologic responses of cancer patients to a variety of infections as well as to mutated cancer antigens. Furthermore, HGF deregulation has been causally associated with tumorigenesis in breast, skin, stomach, prostate, skin, and lung [147,148]. Since fibroblast-specific HGF up-regulation by Tpl2 inhibition was reported in colorectal carcinogenesis [99], potentially enhanced carcinogenesis by activation of HGF-c-Met signaling pathways need to be exercised in the future clinical use of Tpl2 inhibitors in chronic inflammatory diseases [149]. Unfortunately, the role of Tpl2 in tumorigenesis is complex, as either over-expression or reduced-expression can promote tumor formation depending on the cancer type [6]. Therefore, Tpl2 inhibitors may paradoxically lead to the development of secondary malignancies by suppressing anti-tumorigenic mechanisms of Tpl2 kinase in some tissues including skin and lung [94,98]. 

## 10. Conclusions

Anti-cancer targeting agents have major advantages for clinical uses compared with conventional cytotoxic chemotherapeutic agents; cancer specific effects and less systemic toxicities. Since targeted therapeutic agents are developed against specific molecular targets, it is of crucial importance to elucidate optimal target candidates. Tpl2, a MAP3K, participates in a broad range of cancer-related signaling pathways and induces tumorigenesis and progression of many human cancers. Several Tpl2 inhibitors have been developed for immunological disorders; they could be tested for anti-cancer effects using various cancer *in vitro* and *in vivo* models preclinically. Given that Tpl2 signals are cell type-specific as well as stimulus-specific, Tpl2 could be a novel and attractive therapeutic target for many different malignancies with cancer-specific dependence toward Tpl2-mediated oncogenic pathways, such as breast, colorectal, gastric, prostate, and kidney cancer. However, due to the probability of secondary malignancies by Tpl2 targeting, clinical application of Tpl2 as a novel therapeutic target for advanced cancer patients needs to be further validated. 
